# Inexpensive and non-toxic water repellent coatings comprising SiO_2_ nanoparticles and long chain fatty acids

**DOI:** 10.1039/c8ra04707c

**Published:** 2018-07-31

**Authors:** Frances L. Heale, Kristopher Page, James S. Wixey, Philip Taylor, Ivan P. Parkin, Claire J. Carmalt

**Affiliations:** Department of Chemistry, University College London 20 Gordon Street London WC1H 0AJ UK c.j.carmalt@ucl.ac.uk; AzkoNobel Wexham Road, Slough SL2 5DS UK

## Abstract

Special wettability durable coatings, with average water contact angles exceeding 140°, have been fabricated utilising functionalised hydrophobic-SiO_2_ (H-SiO_2_) particles embedded in fatty acids. The inexpensive and non-toxic H-SiO_2_ particles imparted delicate lotus leaf inspired hierarchical surface nano-morphologies while the fatty acid modification afforded a suitable drop in surface energy. Comparison studies were carried out to explore the effects of fatty acid chain length and pipette as opposed to spray coating deposition methods on the coatings hydrophobicity. It was determined that the longest chain length fatty acid coatings showed enhanced hydrophobic properties due to their extended hydrophobic alkyl chain. A pipette deposited suspension containing H-SiO_2_ nanoparticles and octadecanoic acid generated a coating with the most favourable average water contact and tilting angles of 142 ± 6° and 16 ± 2° respectively.

## Abstract 2

Special wettability durable coatings, with water contact angles exceeding 140°, have been fabricated using inexpensive and non-toxic functionalised hydrophobic-silica nanoparticles embedded in fatty acids.

## Graphical abstract

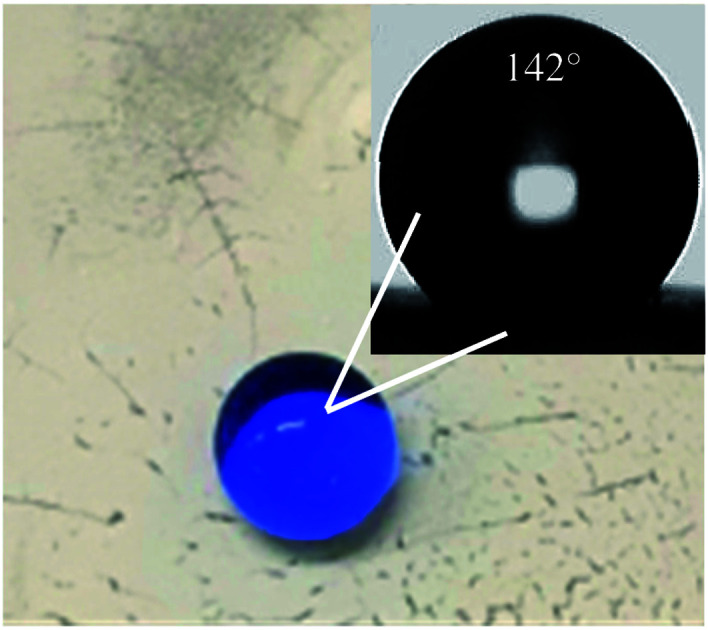

## Introduction

Water retardant ‘smart’ materials^1^ are central to a plethora of novel applications, such as anti-stick surfaces for microdevices, microfluidics,^2^ oil–water separation^3^ and self-cleaning glasses/coatings/textiles.^4,5^ The functional properties of these materials can be understood using Neinhuis and Barthlott's description of the lotus effect – named after naturally superhydrophobic (average water contact angle > 150°) lotus plant leaves.^6^ The pair determined that the leaves' intricate surface micro/nano-morphology combined with their low surface energy coating cause water droplets to reside in a near spherical form on the anti-wetting surface.^6^ Consequently, many artificial superhydrophobic surfaces use biomimicry to recreate this extreme wetting phenomenon.^6–9^

Neinhus and Barthlott's work has inspired many biomimetic superhydrophobic surfaces found in the literature. Plasma etching,^10,11^ lithography,^12,13^ switchable electrochemical deposition,^14,15^ micro-phase separation,^14^ templating,^16,17^ nanoparticles assembly and nano-fabrication^18,19^ are several of the commonly used synthesis routes. These approaches often involve time intensive multistep fabrication pathways that are unsuitable for large scale commercial coating production. From this, Lu *et al.* used a facile method to produce robust paints from nanoscale TiO_2_ particles and a £6 per gram perfluorooctyltriethoxysilane,^20^ fluoro-containing SiO_2_ nanoparticles were synthesised by Wang's research team^21^ and Liang *et al.* used a slightly more involved procedure to create alkenyl-functionalized SiO_2_ particles which were grafted and co-cast with a fluoroalkylsilane.^22^ Additional work in this field includes: transparent superhydrophobic SiO_2_ paper generated using octadecyltrichlorosilane functionalised nanoparticles,^23^ insulating silica aerogels fabricated from the one-step drying of polyethoxydisiloxane/methyltrimethoxysilane based sols^24^ and Shi *et al.* fabricated a highly water repellent SiO_2_/polyvinylidene fluoride film *via* spray coating.^25^ Whilst extremely functional (average water contact angles 140–174°), each of these surfaces are still flawed – this time by material expense and toxicity.

The hydrophobic properties of the cheaper, more environmentally friendly and non-fluorinated octadecanoic acid (£25 per kilogram) have been explored on chemically etched zinc, aluminium or glass substrates. Wei, Chen and Mittal independently generated hierarchical roughness by immersing their respective surfaces in concentrated HCl. A final coating of octadecanoic acid sufficiently lowered their surface energies. Average water contact angles > 150° were achieved in all cases but unfortunately substrate etching substantially reduced the versatility of said methods.^26–28^ To improve on many existing approaches, extreme wetting regimes should be afforded after one treatment of any substrate using non-fluorinated economically viable coating precursors.

**Fig. 1:**
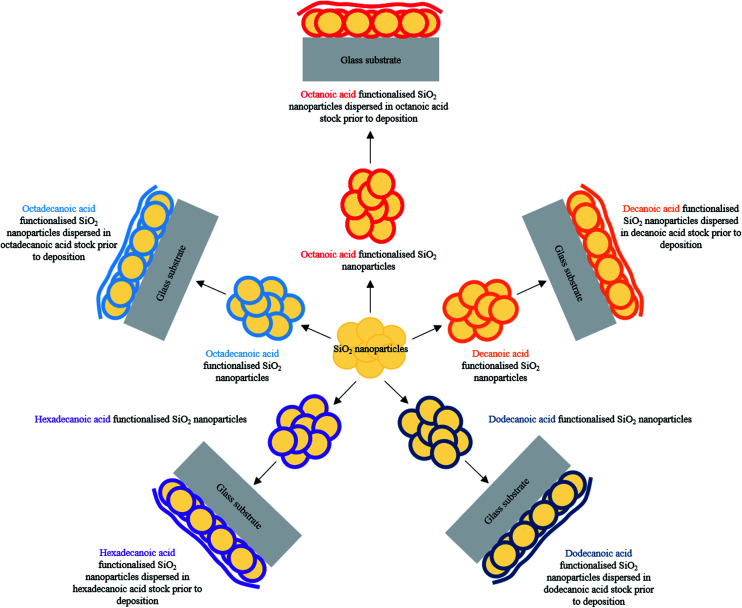
Schematic displaying SiO_2_ nanoparticle functionalisation stages for each fatty acid chain length: octanoic acid (C_8_), decanoic acid (C_10_), dodecanoic acid (C_12_), hexadecanoic acid (C_16_) and octadecanoic acid (C_18_).

A facile production of inexpensive, non-toxic water repellent surface coatings involving a one pot method is described herein. Surface structuring functionalised SiO_2_ nanoparticles were combined with low surface energy fatty acids^29–31^ (C_8_–C_18_ carbon chain lengths) to establish the desired lotus-like effect upon curing, Table 1. Careful optimisation of hydrophobic-SiO_2_ (H-SiO_2_) particle loading, fatty acid concentration and chain length and coating deposition method afforded comparably high average water contact angles on octadecanoic acid coatings, Fig. 1. Clear trends indicated that improved water repellency was associated with coatings containing long chain acids and, in some cases, matched the functionality of fluorinated alternatives.

In addition to the high average water contact angles, the Cassie–Baxter effect explained why the coatings also showed relatively low average water tilting angles.^32^ This wetting state allowed water to remain suspended on top of an air layer entrapped between surface asperities.^33^ Subsequently, liquid droplets rolled from the material collecting dust and dirt particles; an action that rendered the surface self-cleaning.^20,34–36^ More recently these single application non-fluorinated coatings^37–41^ have generated interest from the coatings industry as the long chain acids suitably fulfil the low surface energy hydrophobicity requirement, are low cost, have marketable viability and maintain performance. Therefore, fine tuning this facile one-pot method could potentially result in compatibility with commercial self-cleaning products.^42,43^

## Experimental

### Materials

Unrefined SiO_2_ particles (0.5–1.0 μm diameter) and fatty acids were purchased from Sigma-Aldrich, AEROSIL® OX50 SiO_2_ nanoparticles were acquired from Evonik and laboratory solvents were bought from Fisher Scientific. All chemicals were of analytical standard and were used as received.

### Fabrication of hydrophobic-SiO_2_ particles (H-SiO_2_)

Octanoic (C_8_H_16_O_2_), decanoic (C_10_H_20_O_2_), dodecanoic (C_12_H_24_O_2_), hexadecanoic (C_16_H_32_O_2_) and octadecanoic acids (C_18_H_36_O_2_) (2.00 wt%) were separately stirred in different aliquots of absolute ethanol (88.00 wt%), 40 min at 40 °C, prior to the addition of innately hydrophilic SiO_2_ nanoparticles (10.00 wt%). After a further 20 min of stirring, the five SiO_2_ particle containing suspensions were oven dried at 60 °C for 120 min. This process afforded hydrophobic-SiO_2_ (H-SiO_2_) particles coated in selected non-fluorinated hydrocarbon chains.

### Fabrication of hydrophobic slurries

Hydrophobic-SiO_2_ (H-SiO_2_) nanoparticles were sonicated, 60 min at 40 °C, in their respective octanoic, decanoic, dodecanoic, hexadecanoic or octadecanoic acid/ethanol mixture. In every case, H-SiO_2_ particles had been treated with the corresponding polymer material in which they were finally dispersed. Optimised particle loadings and acid concentration compositions are outlined in Table 1.

**Table 1:** Optimised hydrophobic-SiO_2_ (H-SiO_2_) particle, fatty acid and solvent loadings for water repellent coating slurries. SiO_2_ nanoparticles (10.00 wt%) were pre-functionalised in their respective fatty acid (2.00 wt%)/ethanol (88.00 wt%) mixture

Fatty acid	Carbon chain length	H-SiO_2_ nanoparticle loading/wt%	Fatty acid loading/wt%	Ethanol loading/wt%
Octanoic	8	6.00 ± 0.01	2.44 ± 0.01	91.56 ± 0.01
Decanoic	10	6.00 ± 0.01	1.94 ± 0.01	92.06 ± 0.01
Dodecanoic	12	6.00 ± 0.01	4.52 ± 0.01	89.48 ± 0.01
Hexadecanoic	16	6.00 ± 0.01	5.79 ± 0.01	88.21 ± 0.01
Octadecanoic	18	6.00 ± 0.01	3.21 ± 0.01	90.79 ± 0.01

### Coating application method

Glass substrates were covered in double sided Scotch tape (25 × 30 mm) to aid coating adhesion. Pipette application (1 mL of coating material deposited in the centre of the taped region using a Pasteur pipette) and spray coating (5 s duration, zigzag motion, ∼10 cm distance from substrate) were the two methods utilised to deposit hydrophobic slurries onto the taped surfaces. Whilst octadecanoic acid containing samples were dried at 60 °C for 20 min to prevent recrystallisation, all other coatings were dried overnight at room temperature and pressure. Spray coating was carried out using a BADGER airbrush spray gun and SprayCraft universal airbrush propellant.

### Characterisation

X-ray photoelectron spectroscopy (XPS) was carried out using a Thermo Scientific XPS K-Alpha X-ray Photoelectron Spectrometer with a monochromated Al Kα X-ray source at 1486.6 eV. Atmospheric pressure thermogravimetric analysis (TGA) was carried out using a Netzsch Jupiter analyser. Fourier transform infra-red (FT-IR) spectroscopy was performed using Bruker alpha platinum-ATR equipment (650 to 4000 cm^−1^). Transmission electron microscopy (TEM) was completed using 100 kV JEOL CX100 equipment to determine unrefined and functionalised SiO_2_ particle sizes. Surface topographies were investigated using a JEOL JSM-6301F scanning electron microscope (SEM) with an acceleration voltage of 5 or 10 kV.

### Functional testing

Three water contact angles were measured per coating at ambient temperature *via* the sessile-drop method using a FTA 100 optical contact angle meter (5 μL water droplet). An average value and associated error were calculated for each sample. The tilting angle, defined as the angle at which a water droplet readily slides off a slanted surface (fixed droplet volume of 0.5 mL), was recorded using a digital angle finder. Averages and standard deviations were calculated.

A high-speed camera (fps1000HD-256 made by The Slow Motion Camera Company Ltd., Hertfordshire, UK) was used to capture methylene blue dyed water droplets bouncing on the functional surfaces to confirm water repellency. Samples were also immersed in vegetable oil (20 s) prior to further water contact angle tests for coating robustness comparison.

## Results and discussion

A one pot method was developed to superhydrophobic SiO_2_ coatings from functionalised hydrophobic-SiO_2_ (H-SiO_2_) nanoparticles embedded in fatty acids. H-SiO_2_ particles were produced by stirring SiO_2_ nanoparticles in a fatty acid (octanoic, decanoic, dodecanoic, hexadecanoic and octadecanoic acid)/ethanol mixture. The H-SiO_2_ slurries were prepared by sonicating H-SiO_2_ particles in their respective octanoic, decanoic, dodecanoic, hexadecanoic and octadecanoic acids stock solutions, Fig. 1. Resulting white, opaque coatings remained adhered to double-sided tape covered microscope slide without peeling after a 6 month period of storage at room temperature and pressure.

**Fig. 2:**
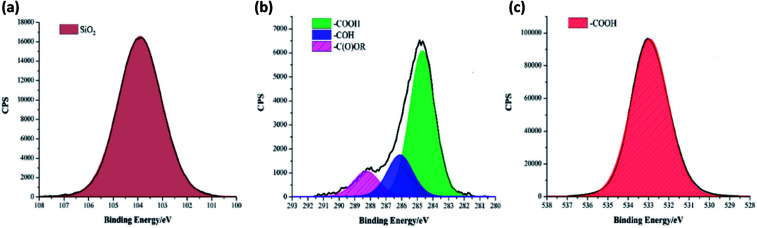
Deconvoluted XPS (a) Si2p, (b) C1s and (c) O1s scans of the coating comprising octadecanoic acid functionalised SiO_2_ nanoparticles (6.00 wt%) originally in an octanoic acid (3.21 wt%)/ethanol (90.79 wt%) mixture.

**Fig. 3:**
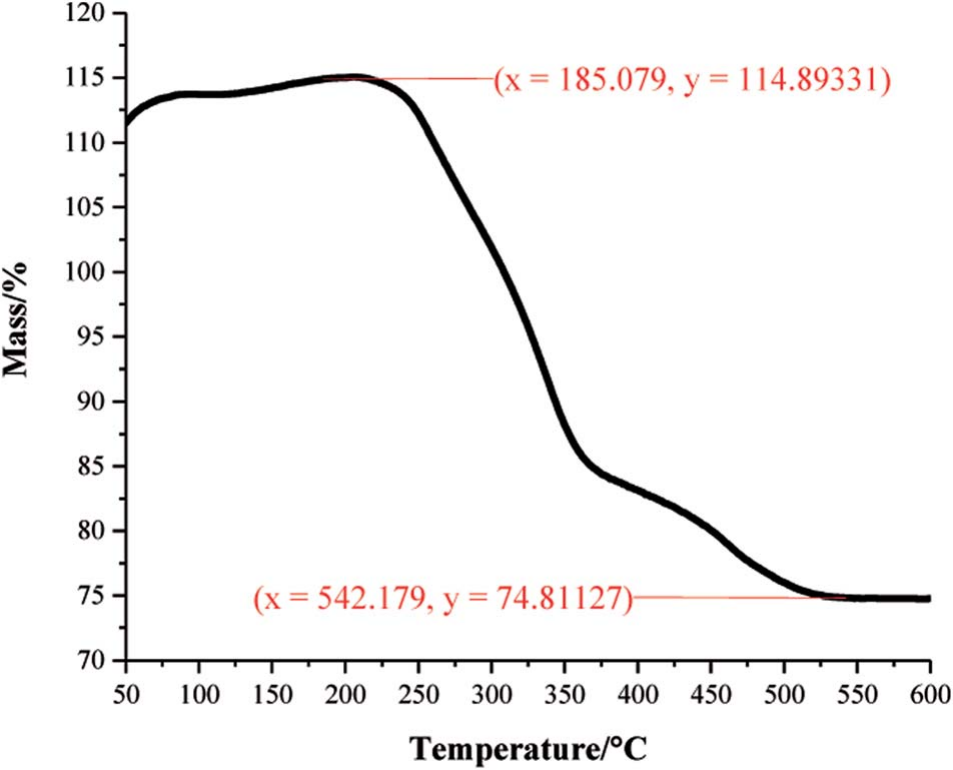
Thermogravimetric analysis (TGA) of the coating comprising octadecanoic acid functionalised SiO_2_ nanoparticles (6.00 wt%) in a dried octadecanoic acid (3.21 wt%)/ethanol (90.79 wt%) mixture. The percentage mass change was plotted against increasing temperature to determine the amount of coating material surrounding the particles. Mass percentage rose above 100% due to unavoidable air buoyancy effects.

XPS data was used to determine the chemical environments found in the acid samples containing embedded functionalised SiO_2_ nanoparticles. Resulting data allowed fatty acid/particle binding method determination. The octadecanoic acid coating, seen in Fig. 2, displays modelled Si2p, C1s and O1s scans which confirmed the presence of acid, alcohol and ester groups. The peak at 103.9 eV in the Si2p scan established that SiO_2_ particles were present at the surface of the sample. The C1s scan closely matched environments identified in the octanoic acid functionalised SiO_2_ starting material; 284.9 eV (C–O(OH) environment),^3^ 286.8 eV (C–OH environment)^4^ and 289.1 eV (C–O(OR) environment).^44^ Peaks in the O1s scan further supported the presence of ester linkages between SiO_2_ particles and the fatty acid. Consistency in acid/particle linkage was supported by the acid, alcohol and ester environments which were reported in all hydrophobic coatings, irrespective of fatty acid chain length. Furthermore, Fig. 3 presents the thermogravimetric analysis (TGA) collected for H-SiO_2_ nanoparticles functionalised with the long chain octadecanoic acid coating, air buoyancy effects gave rise to a mass percentage greater than 100% at 50 °C. From this, it was determined that the organic fatty acid mass loss occurred at temperatures between 200 and 600 °C. It is most probable that the organic material removed from the sample at temperatures nearing 600 °C were chemically bonded to the nanoparticles' surface as a significant amount of thermal energy was required for removal. Any additional acid material capped the functionalised particles by secondary forces, as represented by the mass loss at lower temperatures.

**Fig. 4:**
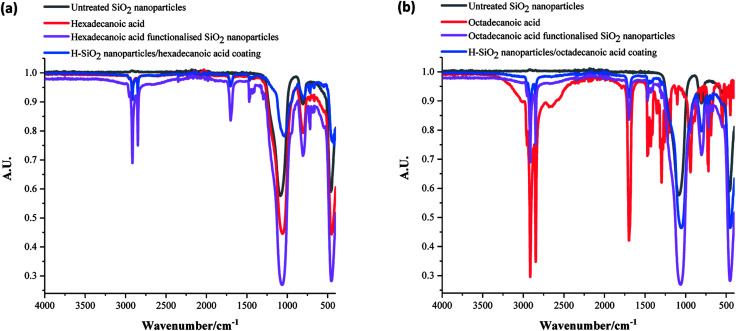
Fourier transform infrared (FT-IR) spectra of (a) hexadecanoic acid functionalised SiO_2_ (H-SiO_2_) particles (6.00 wt%) in hexadecanoic acid (5.79 wt%)/ethanol (88.21 wt%) mixture and (b) octadecanoic acid functionalised SiO_2_ (H-SiO_2_) particles (6.00 wt%) in octadecanoic acid (3.21 wt%)/ethanol (90.79 wt%) mixture. SiO_2_ and acid precursors also plotted.

The functional groups in uncoated SiO_2_ nanoparticles, all acid precursors, acid functionalised SiO_2_ nanoparticles and the coatings with H-SiO_2_ nanoparticles were then compared using FT-IR analysis. Fig. 4 displays the spectra achieved on samples containing some of the longer chain acids. The strongest –CH_2_ symmetric alkane stretches and C=O carboxylic acid stretches were detected in both the hexadecanoic and octadecanoic acid precursors at around 2850 cm^−1^ (sh, w) and 1700 cm^−1^ (sh, w) respectively.^1^ Other peaks at 1060 cm^−1^ (br, m), 770 (sh, m) and 760 (sh, w), originally seen in the H-SiO_2_ spectra, represented Si–O–Si asymmetric transverse-optical stretching, symmetric Si–O–Si stretching and bending respectively.^45^ An absence of the broad O–H stretch at 3000 cm^−1^ was typical of acid dimerization in all samples.^2^ Peak positions showed no significant deviation with fatty acid chain length as chemical properties were similar.

**Fig. 5:**
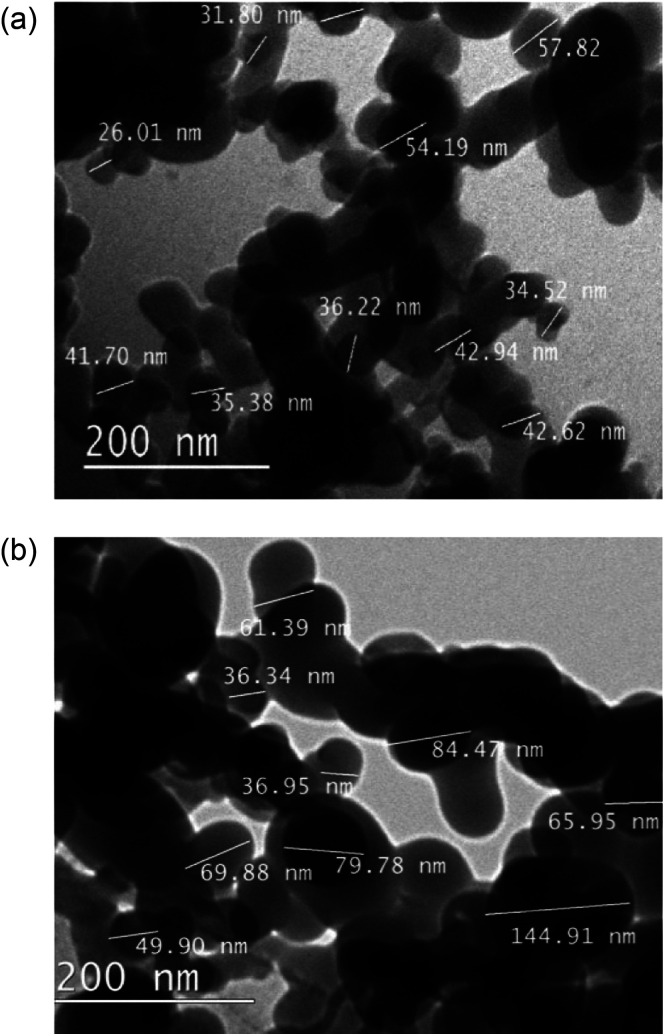
Transmission electron microscope (TEM) size determination of (a) SiO_2_ nanoparticles and (b) octadecanoic acid functionalised SiO_2_ (H-SiO_2_) particles.

Transmission electron microscope (TEM) images of the as received nanoparticles, Fig. 5, suggested particle diameters were <60 nm; the smallest recorded were 25 nm. After functionalisation with fatty acids, particle sizes were substantially increased, ∼100 nm. The correlation between H-SiO_2_ particle diameters and final surface morphology required the use of these small scale precursors to ensure some coating nanostructure was achieved.

**Fig. 6:**
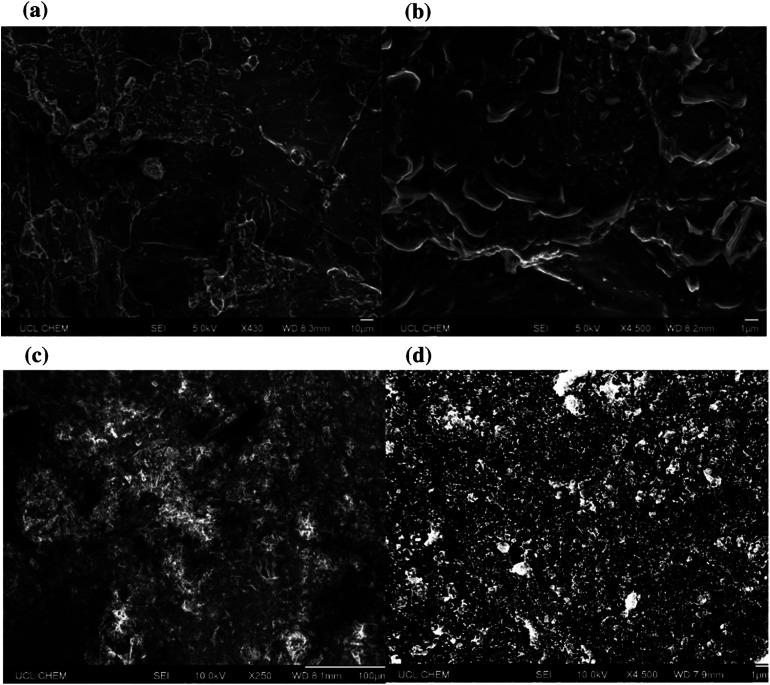
High magnification SEM images (a) and (b) depict the topography of the coating containing octanoic acid functionalised SiO_2_ particles (H-SiO_2_) (6.00 wt%) in octanoic acid (2.44 wt%)/ethanol (91.56 wt%) mixture. SEM images (c) and (d) depict the topography of the coating containing octadecanoic acid functionalised SiO_2_ particles (H-SiO_2_) (6.00 wt%) in octadecanoic acid (3.21 wt%)/ethanol (90.79 wt%) mixture. All samples were gold sputtered to minimise the effects of charging.

Surface topographies were then assessed using scanning electron microscopy (SEM), Fig. 6. Nanoscale protrusions and areas of particle agglomeration (>1 μm) were detected under high magnification. Whilst the presence of micro clumps was not an issue for the surfaces that contained longer chain length acids, the analysis highlighted that more extreme particle clumping was an issue for the shorter chain samples. In the case of octanoic acid the shortened hydrophobic chain meant that any large-scale clumps would have more heavily compromised hydrophobicity.

### Functional testing

**Fig. 7:**
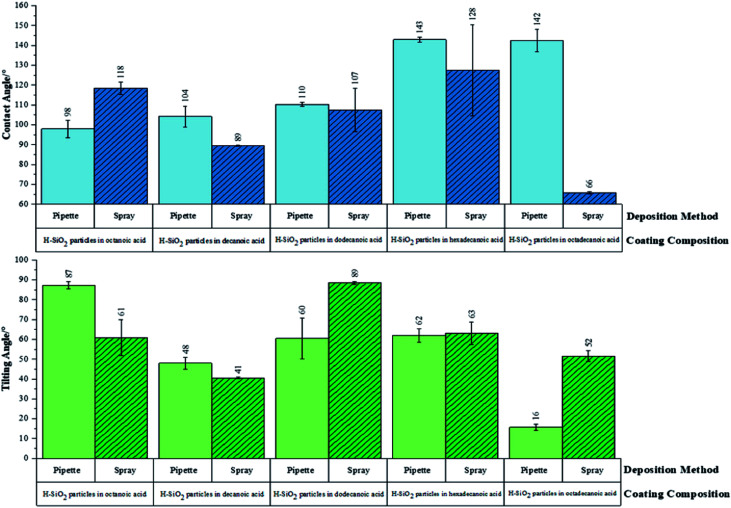
Average contact and tilting angle data for optimised fatty acid coatings containing the preferred hydrophobic functionalised SiO_2_ (H-SiO_2_) particle loading. H-SiO_2_ (6.00 wt%)/octanoic acid (2.44 wt%)/ethanol (91.56 wt%), H-SiO (6.00 wt%)/decanoic acid (1.94 wt%)/ethanol (92.06 wt%), H-SiO_2_ (6.00 wt%)/dodecanoic acid (4.52 wt%)/ethanol (89.48 wt%), H-SiO_2_ (6.00 wt%)/hexadecanoic acid (5.79 wt%)/ethanol (88.21 wt%), and H-SiO_2_ (6.00 wt%)/octadecanoic acid (3.21 wt%)/ethanol (90.79 wt%) coatings were separately applied to prepared glass microscope slides *via* the pipette or spray coating method.

Initially, functional testing was carried out on untreated/as received SiO_2_ nanoparticles. This precursor was deemed superhydrophilic in nature as average water contact angles were <5°. Surface wettability was subsequently determined for the optimised water repellent coatings. Fig. 7 proves the hydrophobicity of pipette *versus* spray coated samples were similar. The coating sample containing hexadecanoic acid had average water contact angles of 142 ± 1° and 128 ± 23° for pipette and spray application respectively. Average tilting angles were found to be indistinguishable within experimental error.

The largest difference in hydrophobicity was realised when the octadecanoic acid polymer was incorporated into coating slurries; the pipette application generated an average contact angle ∼80° larger and an average tilting angle ∼40° lower than the spray application alternative. In contrast, the functional results were improved by ∼30° on the sprayed short chain decanoic acid coatings. This data confirmed that the use of spray deposition benefited short chain polymer systems by distributing H-SiO_2_ particles more evenly in the less viscous shorter chain acids; short chain acids showed no sign of crystallising during this process. With that said, pipetting was advantageous for the more bulky octadecanoic acid coatings where a maximum average water contact angle of 142 ± 6° was achieved. The longer chain acids, such as octadecanoic acid, had a greater tendency to crystallise during slurry deposition. It was found that crystallisation during pipette application was reduced due to the speed and nature of deposition (reaction temperature was closely maintained throughout). This promoted even surface coverage and likely elevated average water contact angles; unfortunately this was not the case for the spray deposition alternative as the method promoted slurry cooling. In spite of this, the environmentally friendly and cheap ‘hydrophobic coatings’ are of significance as they have only been marginally outperformed by coatings of much greater toxicity and expense. Sino *et al.* created a fluoroalkylsilane based emulsion with TiO_2_/ZnO particles (water contact angle, >150°) while other work documents the use of TiO_2_/SiO_2_ particles combined with fluorinated polymers and epoxy resins (water contact angle, ∼152°).^46,47^

**Fig. 8:**
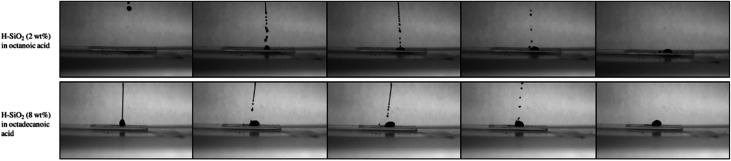
High-speed camera images of water droplets falling onto the coating containing octanoic acid functionalised SiO_2_ particles (H-SiO_2_) (6.00 wt%) originally in an octanoic acid (2.44 wt%)/ethanol (91.56 wt%) mixture and the coating containing octadecanoic acid functionalised SiO_2_ nanoparticles (H-SiO_2_) (6.00 wt%) originally in an octadecanoic acid (3.21 wt%)/ethanol (90.79 wt%) mixture.

A high-speed camera was used to support the average water contact angle data results obtained on the shortest and longest fatty acid chain length coatings. Fig. 8 presents snap shots of water droplets being pipetted onto the optimised coating surfaces. In the case of structured octanoic acid samples, water droplets landed and remained pinned to the coating with marginal surface repulsion. When contrasted with the roughened octadecanoic acid surface, small water droplets bounced up to three times on the functionalised material before resting in a more spherical form. This result improves upon the ∼2 bounces seen on a similarly water retardant but ‘harder’ surface fabricated by Crick *et al.*^48^ Crick's work made use of SiO_2_ particles modified with the expensive and environmentally harmful polydimethylsiloxane (PDMS).^48^

**Fig. 9:**
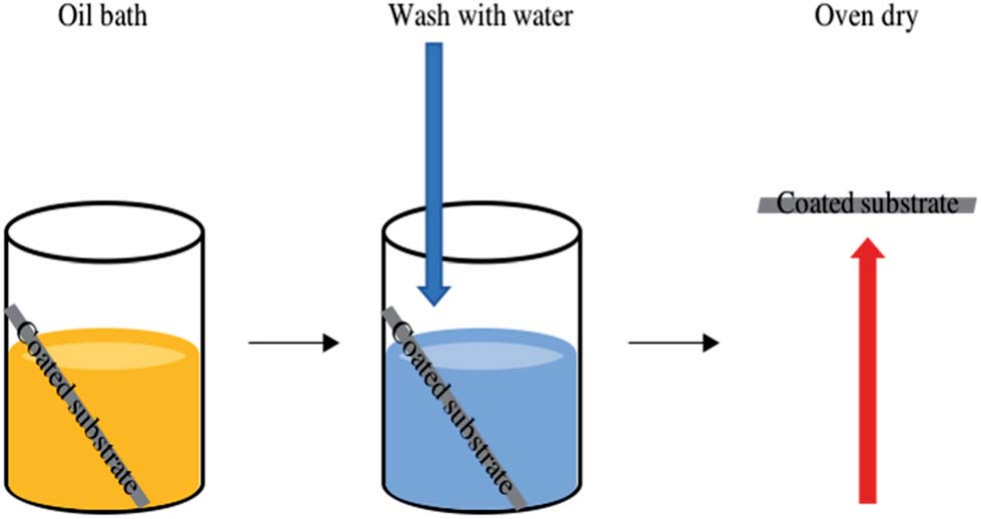
Optimised octanoic, decanoic, dodecanoic, hexadecanoic and octadecanoic acid based coatings containing embedded fatty acid functionalised SiO_2_ particles were subjected to the oil bath test. Sample robustness was monitored by immersing the coatings in vegetable oil prior to washing with water and oven drying.

The functionality of the SiO_2_/fatty acid coatings, prepared in this work, were preserved after the oil immersion test, Fig. 9. The samples showed exceptional durability after being submerged in vegetable oil, washed in water and oven dried. In all cases the average water contact angle remained unaltered within experimental error. For example, the octadecanoic acid containing coating had an average water contact angle of 134 ± 11° after the oil bath test and the tilting angles were comparable.

### Coating performance

Consistent chemical properties present in all fatty acid coatings resulted in near identical XPS, FT-IR, TEM and SEM data irrespective of carbon chain length. In contrast, differences arose when comparing sample functionality. The pipette deposit of H-SiO_2_ nanoparticles in the long chain octadecanoic acid coating afforded the highest average water contact angle (∼142°) whereas the short chain octanoic acid with embedded nanoparticles was considerably lower (∼111°). This observation was justified by considering the nonpolar –(CH_2_)_*n*_– to polar –COOH group ratio; as the carbon chain length increased so does the net repulsion between the hydrophobic nonpolar aliphatic chain and surface water. The hydrophobic character of the long chain easily dominated, negating the polar influence of the acid functional group that permits hydrogen bonding with water.^49^

## Conclusion

We have successfully generated inexpensive and non-toxic coatings containing functionalised SiO_2_ particles and long carbon chain length fatty acids. These durable hydrophobic coatings have been achieved using a facile one pot synthesis followed by pipette or spray deposition methods. Trends suggested that water repellency was increased with fatty acid carbon chain length; the coating comprising hydrophobic-SiO_2_ (H-SiO_2_) particles (6.00 wt%) originally in an octadecanoic acid (3.21 wt%)/ethanol mixture (90.79 wt%) had favourable average water contact and tilting angles of 142 ± 6° and 16 ± 2° respectively. Further work should be aimed at scaling up this process by making use of dip coating techniques or by incorporating these slurries into commercial products to form the ‘smartest’ self-cleaning surfaces.

## Conflicts of interest

There are no conflicts to declare.

## Supplementary Material

## References

[cit1] Zhang Y., Wei S., Liu F., Du Y., Liu S., Ji Y., Yokoi T., Tatsumi T., Xiao F.-S. (2009). Nano Today.

[cit2] Vinogradova O. I., Dubov A. L. (2012). Mendeleev Commun..

[cit3] Zhu H., Chen D., Li N., Xu Q., Li H., He J., Lu J. (2017). Appl. Catal., B.

[cit4] Afzal S., Daoud W. a., Langford S. J. (2014). J. Mater. Chem. A.

[cit5] Nine M. J., Cole M. A., Johnson L., Tran D. N. H., Losic D. (2015). ACS Appl. Mater. Interfaces.

[cit6] Barthlott W., Neinhuis C. (1997). Planta.

[cit7] Liu Y., Chen X., Xin J. H. (2008). Bioinspiration Biomimetics.

[cit8] Byun D., Hong J., Saputra, Ko J. H., Lee Y. J., Park H. C., Byun B. K., Lukes J. R. (2009). J. Bionic Eng..

[cit9] Zheng Y., Gao X., Jiang L. (2007). Soft Matter.

[cit10] Durret J., Szkutnik P., Frolet N., Labau S., Gourgon C. (2018). Appl. Surf. Sci..

[cit11] Teisala H., Tuominen M., Kuusipalo J. (2014). Adv. Mater. Interfaces.

[cit12] Feng J., Tuominen M. T., Rothstein J. P. (2011). Adv. Funct. Mater..

[cit13] Balu B., Breedveld V., Hess D. W. (2008). Langmuir.

[cit14] Ma M., Hill R. M. (2006). Curr. Opin. Colloid Interface Sci..

[cit15] Zhang X., Shi F., Yu X., Liu H., Fu Y., Wang Z., Jiang L., Li X. (2004). J. Am. Chem. Soc..

[cit16] Peng C. W., Chang K. C., Weng C. J., Lai M. C., Hsu C. H., Hsu S. C., Li S. Y., Wei Y., Yeh J. M. (2013). Polym. Chem..

[cit17] Dislaki E., Pokki J., Pané S., Sort J., Pellicer E. (2018). Appl. Mater. Today.

[cit18] Wang Y., Shi Y., Pan L., Yang M., Peng L., Zong S., Shi Y., Yu G. (2014). Nano Lett..

[cit19] Rahmawan Y., Xu L., Yang S. (2013). J. Mater. Chem. A.

[cit20] Lu Y., Sathasivam S., Song J. L., Crick C. R., Carmalt C. J., Parkin I. P. (2015). Science.

[cit21] Wang H., Fang J., Cheng T., Ding J., Qu L., Dai L., Wang X., Lin T. (2008). Chem. Commun..

[cit22] Liang J., Wang L., Bao J., He L. (2016). Colloids
Surf., A.

[cit23] Li J., Wan H., Ye Y., Zhou H., Chen J. (2012). Appl. Surf. Sci..

[cit24] Zhu X., Naz H., Ali R. N., Yang Y., Zheng Z., Xiang B., Cui X. (2018). Mater. Res. Express.

[cit25] Shi Y., Xiao X. (2016). J. Dispersion Sci. Technol..

[cit26] Chen C., Yang S., Liu L., Xie H., Liu H., Zhu L., Xu X. (2017). J. Alloys Compd..

[cit27] Wei Z., Jiang D., Chen J., Ren S., Li L. (2017). J. Adhes. Sci. Technol..

[cit28] Mittal T., Tiwari S., Tiwari S. K. (2017). J. Coat. Technol. Res..

[cit29] Joseph K. R., Neto C. (2010). Aust. J. Chem..

[cit30] Varesano A., Rombaldoni F., Tonetti C. (2013). Fibers Polym..

[cit31] Wan Y., Wang Z., Xu Z., Yu L., Qi C. (2011). Thin Solid Films.

[cit32] Cassie A. B. D., Baxter S. (1944). Trans. Faraday Soc..

[cit33] Simpson J. T., Hunter S. R., Aytug T. (2015). Rep. Prog. Phys..

[cit34] Xue C., Li Y., Zhang P., Ma J., Jia S. (2014). ACS Appl. Mater. Interfaces.

[cit35] Surekha K. and Sundararajan S., Self-cleaning glass, Elsevier Ltd, 2015

[cit36] Parkin I. P., Palgrave R. G. (2005). J. Mater. Chem..

[cit37] Zhang X., Geng T., Guo Y., Zhang Z., Zhang P. (2013). Chem. Eng. J..

[cit38] Xue C. H., Zhang Z. D., Zhang J., Jia S. T. (2014). J. Mater. Chem. A.

[cit39] Baba E. M., Cansoy C. E., Zayim E. O. (2016). Prog. Org. Coat..

[cit40] Qing Y., Zheng Y., Hu C., Wang Y., He Y., Gong Y., Mo Q. (2013). Appl. Surf. Sci..

[cit41] Kim H. K., Cho Y. S. (2015). Colloids Surf., A.

[cit42] Taheri M., jahanfar M., ogino K. (2017). Surf. Interfaces.

[cit43] Xue L., Yang X., Li Z., Sun Y., Feng P., He Y., Qu Z., Dai T., Zhang J.-G., Qin T., Xu J., Zhang W. (2018). Sol. Energy Mater. Sol. Cells.

[cit44] Beamson G. and Briggs D., High Resolution XPS of organic polymers, the scienta ESCA 300 database, John Wiley & Sons, 1992, vol. 15

[cit45] Socrates G. , Infrared and Raman characteristic group frequencies, John Wiley & Sons, 2004

[cit46] Sino P. A. L., Herrera M. U., Balela M. D. L. (2017). IOP Conf. Ser.: Mater. Sci. Eng..

[cit47] Wu Y., Jia S., Wang S., Qing Y., Yan N., Wang Q., Meng T. (2017). Chem. Eng. J..

[cit48] Crick C., Parkin I. P. (2011). Chem. Commun..

[cit49] Smith R., Tanford C. (1973). Proc. Natl. Acad. Sci. U. S. A..

